# COVID-19 prevalence, symptoms, and sociodemographic disparities in infection among insured pregnant women in Northern California

**DOI:** 10.1371/journal.pone.0256891

**Published:** 2021-09-03

**Authors:** Jennifer L. Ames, Assiamira Ferrara, Lyndsay A. Avalos, Sylvia E. Badon, Mara B. Greenberg, Monique M. Hedderson, Michael W. Kuzniewicz, Yinge Qian, Kelly C. Young-Wolff, Ousseny Zerbo, Yeyi Zhu, Lisa A. Croen

**Affiliations:** 1 Division of Research Kaiser Permanente Northern California, Oakland, CA, United States of America; 2 Department of Obstetrics and Gynecology, East Bay, Kaiser Permanente Northern California, Oakland, CA, United States of America; SUNY Downstate: SUNY Downstate Health Sciences University, UNITED STATES

## Abstract

**Background:**

Research on COVID-19 during pregnancy has mainly focused on women hospitalized for COVID-19 or other reasons during their pregnancy. Little is known about COVID-19 in the general population of pregnant women.

**Objective:**

To describe the prevalence of COVID-19, symptoms, consequent healthcare use, and possible sources of COVID-19 exposure among a population-based sample of pregnant women residing in Northern California.

**Methods:**

We analyzed data from 19,458 members of Kaiser Permanente Northern California who were pregnant between January 2020 and April 2021 and responded to an online survey about COVID-19 testing, diagnosis, symptoms, and their experiences during the COVID-19 pandemic. Medical diagnosis of COVID-19 during pregnancy was defined separately by self-report and by documentation in electronic health records (EHR). We examined relationships of COVID-19 with sociodemographic factors, underlying comorbidities, and survey measures of COVID-19-like symptoms, consequent healthcare utilization, and possible COVID-19 exposures.

**Results:**

Among 19,458 respondents, the crude prevalence of COVID-19 was 2.5% (n = 494) according to self-report and 1.4% (n = 276) according to EHR. After adjustment, the prevalence of self-reported COVID-19 was higher among women aged <25 years compared with women aged ≥35 years (prevalence ratio [PR], 1.75, 95% CI: 1.23, 2.49) and among Hispanic women compared with White women (PR, 1.91, 95% CI: 1.53, 2.37). Prevalence of self-reported COVID-19 was higher among women affected by personal or partner job loss during the pandemic (PR, 1.23, 95% CI: 1.02, 1.47) and among women living in areas of high vs. low neighborhood deprivation (PR, 1.74, 95% CI: 1.33, 2.27). We did not observe differences in self-reported COVID-19 between women with and without underlying comorbidities. Results were similar for EHR-documented COVID-19. Loss of smell or taste was a unique and common symptom reported among women with COVID-19 (42.3% in self-reported; 54.0% in EHR-documented). Among women with symptomatic COVID-19, approximately 2% were hospitalized, 71% had a telehealth visit, and 75% quarantined at home. Over a third of women with COVID-19 reported no known exposure to someone with COVID-19.

**Conclusions:**

Observed COVID-19 prevalence differences by sociodemographic and socioeconomic factors underscore social and health inequities among reproductive-aged women. Women with COVID-19 reported unique symptoms and low frequency of hospitalization. Many were not aware of an exposure to someone with COVID-19.

## Introduction

Pregnant women may be more susceptible to severe COVID-19 illness [1–3], in part due to reduced immunity and increased stress during pregnancy. Factors such as chronic stress, underlying inflammatory conditions such as obesity and asthma, belonging to a racial/ethnic minority group, and socioeconomic factors are also known to compound risk of viral infections and exacerbate inflammation during pregnancy [4–6]. Research also suggests that SARS-CoV-2 infection during pregnancy may increase risk of other adverse women’s and neonatal health outcomes [7, 8]. Despite these concerns, research on COVID-19 in pregnant women remains limited. Several studies have presented the clinical characteristics of COVID-19 in pregnant women admitted for delivery [9–15] or for COVID-19 complications [1, 16]. Fewer studies have characterized symptoms of COVID-19 in a general sample of pregnant women, representative of different stages during pregnancy [17–20]. Furthermore, prior studies have primarily focused on infection prevalence across relatively short time windows and have not described healthcare utilization or sources of COVID-19 exposure in pregnant women during the pandemic.

At Kaiser Permanente Northern California (KPNC), a large integrated healthcare delivery system, we conducted a survey among all women who were pregnant at any time during January 2020 to April 2021. The survey was designed to evaluate the frequency of self-reported COVID-19 testing, medical diagnosis, symptoms and related health care utilization. The aims of the present analysis were to a) estimate the crude prevalence of diagnosed COVID-19 and testing frequency for COVID-19 among pregnant women overall and by sociodemographic characteristics, neighborhood factors, and underlying comorbidities, and b) describe frequencies of symptoms, consequent health care utilization, and possible COVID-19 exposures between January 2020 and May 2021.

## Methods

### Study population

The study setting was KPNC, a large integrated healthcare delivery system serving a membership of 4.5 million people, with approximately 30% of the population residing in the San Francisco Bay Area, Sacramento metropolitan area, and the Central Valley [21].

The study population included KPNC members who were ≥18 years old and at any stage of pregnancy between January 1, 2020 and April 28, 2021. At the time the survey was launched (June 22, 2020), eligible women aged 18+ years were either postpartum, having already delivered a liveborn infant between January 1, 2020 and June 22, 2020, or were currently pregnant and at > = 12 weeks of gestation.

### Study procedures

At the initial survey launch, all eligible women were contacted via email and invited to complete a brief, web-based 25-item research survey through REDCap. Every two weeks thereafter, all newly eligible pregnant women were emailed a study invitation. Although we typically identified and invited eligible women during their second trimester, we also invited a small number of women whose eligibility we ultimately identified late in pregnancy or early in postpartum. Women who did not respond to the initial email invitation received email reminders 7 and 14 days after the initial emails were sent. Participants who started but did not complete the survey received two email reminders, at 7 and 14 days after starting the survey. Regardless of whether they completed the first survey, women were again invited by email to complete a second survey, identical to the first, towards the end of their pregnancy (>34 weeks of pregnancy).

### Survey measures

The present study reports findings from women pregnant between January 2020 and April 2021 who completed the survey between June 22, 2020 and May 10, 2021 and focuses on questions about healthcare provider diagnosis of COVID-19, receipt of COVID-19 diagnostic tests and test results, and experience of symptoms previously reported to be associated with COVID-19 [22]. Women reporting symptoms were additionally asked about healthcare utilization resulting from symptoms, and potential exposures from personal contacts who had or likely had COVID-19 and/or from travel in the 2 weeks before the symptoms started. The survey also collected other information about whether the respondents experienced changes to their or their partner’s employment during the pandemic and whether they received nutritional assistance (see **S1 Appendix** for a copy of the full survey).

### Linkage to the Electronic Health Record (EHR)

Women’s information captured in the KPNC EHR before and during pregnancy, including sociodemographic information (e.g., self-reported race/ethnicity), medical diagnoses, delivery date (actual or expected), and COVID-19 diagnostic codes, was linked to the survey data. We examined respondents’ underlying diagnoses of several conditions previously shown to be related to heightened risk of severe COVID-19 in adults [23–27]. These included any diagnoses, ascertained via clinical diagnosis and/or medication use, of allergy, asthma, autoimmune conditions, obesity, diabetes, and hypertension in the two years preceding survey completion. We used current residential address obtained from EHR to link survey responses to data on neighborhood deprivation index (NDI), [28] an indicator of neighborhood-level socioeconomic position that integrates census variables on education, occupation, housing, and income/poverty.

### Ascertainment of COVID-19

We defined clinically-diagnosed COVID-19 during pregnancy in two ways: according to self-reported diagnosis of COVID-19 made by a healthcare provider or according to documentation in the EHR. Women with a self-reported COVID-19 diagnosis responded “Yes” to the survey question “During your pregnancy, did a healthcare provider ever tell you that you have, or likely have, COVID-19 (Coronavirus)?” Women with EHR-documented diagnosis of COVID-19 had a positive polymerase chain reaction (PCR) test for SARS-CoV-2 and/or a diagnostic ICD-10 code for COVID-19 recorded in their EHR between the date of their last menstrual cycle and the delivery date (for postpartum respondents) or the survey completion date (for currently pregnant respondents). The relevant ICD-10 codes are included in the **S2 Appendix**. Women who did not meet either of these diagnostic definitions were included in the no-COVID-19 group. Women who did not have SARS-CoV-2 PCR test results but had diagnostic codes in their EHR for suspected COVID-19, exposure to COVID-19, or COVID-19 disease counseling were considered suspected COVID-19 (n = 4035) and were excluded from the EHR-documented COVID-19 and no-COVID-19 groups. Of note, during the period of March-October 2020, KPNC was predominantly only testing people with symptoms or known exposures, in accordance with CDC recommendations.

KPNC’s Institutional Review Board approved all study procedures and women indicated informed consent prior to participation in the survey.

### Statistical analyses

Chi-square tests were performed to compare the characteristics between women who did and did not complete the survey. The crude prevalence of COVID-19 during pregnancy (according to self-report and according to EHR documentation) was calculated overall and by age, race/ethnicity, and NDI; variables reflecting individual and neighborhood socioeconomic status; and several underlying comorbidities. Generalized linear models were used to obtain crude and adjusted prevalence ratios for COVID-19. All multivariable models adjusted the prevalence ratios for maternal age category, maternal race/ethnicity, and NDI quartile. Seven women with missing age were excluded from all models. Women with missing values for NDI (n = 105) were included in the missing category and retained in the models. As a secondary analysis, we summarized the frequencies of self-reported COVID-19 diagnostic testing and test-positivity by sociodemographic characteristics and underlying comorbidities among women who were pregnant at the time they completed the survey.

We described differences in self-reported COVID-19-like symptoms, consequent healthcare use, and potential exposures to COVID-19 between women with COVID-19 (for each case definition) and women in the no-COVID-19 group using chi-square tests, or Fisher exact tests when cell counts were <5. A p-value <0.05 was used to determine statistical significance in all tests performed. All statistical analyses were conducted using SAS version 9.4 (SAS Institute).

## Results

Between June 22, 2020 and May 10, 2021, a total of 19,458 of 82,482 eligible women (23.6%) completed the survey, 73.8% while pregnant (of which 92.1% were in their second or third trimester) and 26.2% after delivery (mean [SD]: 10 [7] weeks postpartum). Postpartum respondents were pregnant between March 2019 and April 2021, and the majority (98.3%) gave birth between February 2020, when the first identified cases of COVID-19 were reported in California, and August 2020. The majority of all respondents were in their second trimester (68.9%) and a plurality were 30–34 years old (40.0%), White (47.3%), and living in the lowest quartile of neighborhood deprivation (31.3%) (**Table 1**).

**Table 1:** Characteristics of survey respondents, Kaiser Permanente Northern California.

	Survey Respondents^a^ (n = 19458)
	n (%)
**Currently pregnant**	14353 (73.8)
** Trimester at time of survey invitation**	
** **1^st^ trimester	1129 (7.9)
** **2^nd^ trimester	9885 (68.9)
** **3^rd^ trimester	3339 (23.3)
**Postpartum**	5105 (26.2)
**Maternal age**	
<25	817 (4.2)
25–29	3257 (16.7)
30–34	7788 (40.0)
35+	7589 (39.0)
Missing	7 (0.01)
**Maternal race/ethnicity**	
Asian	4543 (23.4)
Black	780 (4.0)
Hispanic	4045 (20.8)
Pacific Islander/American Indian	285 (1.5)
Other/Missing	594 (3.1)
White	9211 (47.3)
**NDI** ^**b**^	
Quartile 1 (≤25%)	6085 (31.3)
Quartile 2 (>25–50%)	5278 (27.1)
Quartile 3 (>50–75%)	4428 (22.8)
Quartile 4 (>75%)	3562 (18.3)
Missing	105 (0.5)
**Insurance Payer**	
Commercial	17997 (92.5)
Government	1173 (6.0)
Unknown	288 (1.5)
**Received benefits from food and nutrition assistance programs**	
Yes	4192 (21.5)
No	15266 (78.5)
**Job loss during pandemic**	
Permanently	612 (3.2)
Temporarily or reduced hours	2582 (13.3)
**Partner job loss during pandemic**	
Permanently	581 (3.0)
Temporarily or reduced hours	2978 (15.3)
**Underlying comorbidities**	
Any underlying comorbidities^c^	10121 (52.0)
Allergy	4720 (24.3)
Asthma	2381 (12.2)
Autoimmune	2757 (14.2)
Diabetes	261 (1.3)
Hypertension	344 (1.8)
Pre-pregnancy BMI^d^	
** **Underweight (BMI<18.5)	315 (1.6)
** **Normal weight (BMI 18.5–24.9)	7952 (40.9)
** **Overweight (BMI 25–29.9)	4999 (25.7)
** **Obese (BMI≥30)	4330 (22.3)

NDI, Neighborhood Deprivation Index.

^a^ Seventy-nine percent of respondents were KPNC members for at least 9 months in each of the two years preceding their survey completion date.

^b^ NDI quartiles were calculated from the NDI distribution among total invited.

^c^ Underlying comorbidities included allergy, asthma, autoimmune condition, diabetes, hypertension, or obesity documented in electronic health record (EHR) in two years preceding survey completion date.

^d^ 1862 women did not have BMI recorded in the two years before their pregnancy.

In comparison to survey non-respondents, respondents were more likely to be White, ≥30 years old, and live in neighborhoods with low deprivation. The frequency of any history of EHR-documented COVID-19 diagnosis was similar between respondents and non-respondents (2.3% vs. 2.4%, p = 0.60) (**S1 Table**).

### Prevalence of COVID-19 during pregnancy

A total of 494 respondents (2.5%) reported that a healthcare provider told them that they had or likely had COVID-19 during their pregnancy (**Table 2**). The crude prevalence of self-reported COVID-19 was highest for women <25 years of age (5.1%), Hispanic (4.4%) and Black women (3.1%), and women living in neighborhoods with the highest deprivation (i.e., 4th quartile of NDI) (4.1%) (**Table 2**). After adjustment for other sociodemographic characteristics, the prevalence of self-reported COVID-19 remained significantly higher among the youngest women (<25 vs. ≥35: adj-PR [95% CI]: 1.75 [1.23–2.49] and 25–29 vs. ≥35: 1.60 [1.26–2.03]), women of Hispanic race/ethnicity compared with White women (adj-PR[95% CI]: 1.81 [1.28–2.55]), women impacted by personal or partner job loss or reduced hours during the pandemic compared with women without impacted employment (adj-PR[95% CI]: 1.23 [1.02–1.47]), and women living in the highest quartile vs. lowest quartile of NDI (adj-PR [95% CI]: 1.74 [1.33–2.27]). Other socioeconomic indicators such as health insurance payer and receipt of food assistance were not significantly associated with self-reported COVID-19 after adjustment for covariates. While the crude prevalence of EHR-documented COVID-19 was lower than self-reported COVID-19 (1.4% vs 2.5%), prevalence patterns by age, race/ethnicity, and NDI were similar for both definitions (**Table 2**).

**Table 2:** Self-reported and EHR-documented COVID-19 prevalence by sociodemographic and clinical factors, crude and adjusted prevalence ratios.

	Self-reported COVID-19			EHR-documented COVID-19		
	n (%)^a^	Crude PR (95% CI)	Adj. PR (95% CI)^b^	n (%)^a^	Crude PR (95% CI)	Adj. PR (95% CI)^b^
**Total**	494 (2.5)^c^	--	--	276 (1.4)^d^	--	--
**Maternal age (years)**	p<0.0001^e^			p<0.0001^e^		
<25	42 (5.1)	2.56 (1.83–3.57)	1.75 (1.23–2.49)	30 (3.7)	3.67 (2.42–5.56)	2.17 (1.40–3.35)
25–29	131 (4.0)	2.01 (1.59–2.53)	1.60 (1.26–2.03)	82 (2.5)	2.51 (1.85–3.43)	1.81 (1.32–2.50)
30–34	169 (2.2)	1.08 (0.87–1.35)	1.02 (0.82–1.27)	88 (1.1)	1.13 (0.83–1.53)	1.04 (0.77–1.42)
≥35	152 (2.0)	Ref	Ref	76 (1.0)	Ref	Ref
**Maternal race/ ethnicity**	p<0.0001^e^			p<0.0001^e^		
Asian	101 (2.2)	1.21 (0.95–1.55)	1.25 (0.98–1.59)	36 (0.8)	0.82 (0.56–1.21)	0.85 (0.57–1.24)
Black	24 (3.1)	1.69 (1.11–2.57)	1.37 (0.90–2.11)	13 (1.7)	1.72 (0.97–3.07)	1.40 (0.78–2.51)
Hispanic	178 (4.4)	2.40 (1.95–2.95)	1.91 (1.53–2.37)	124 (3.1)	3.17 (2.42–4.16)	2.42 (1.81–3.22)
Other/Missing	22 (2.5)	1.37 (0.88–2.12)	1.26 (0.81–1.95)	14 (1.6)	1.65 (0.94–2.88)	1.53 (0.88–2.69)
White	169 (1.8)	Ref	Ref	89 (1.0)	Ref	Ref
**Insurance Payer**	P = 0.003^e^			p = 0.47^e^		
Commercial	440 (2.4)	Ref	Ref	252 (1.4)	Ref	Ref
Government	41 (3.5)	1.44 (1.05–1.97)	1.01 (0.74–1.40)	21 (1.8)	1.28 (0.82–1.99)	0.80 (0.51–1.25)
Unknown	13 (4.5)	1.84 (1.07–3.16)	1.61 (0.94–2.77)	3 (1.0)	0.74 (0.24–2.31)	0.65 (0.21–2.01)
**Personal/Partner Job loss**	p = 0.0004^e^			p = 0.009^e^		
No	315 (2.3)	Ref	Ref	176 (1.3)	Ref	Ref
Job loss (permanently, temporarily, or hours reduced)	179 (3.2)	1.38 (1.16–1.68)	1.23 (1.02–1.47)	100 (1.8)	1.38 (1.09–1.77)	1.19 (0.93–1.52)
**Received food assistance**	p<0.0001^e^			p<0.0001^e^		
No	354 (2.3)	Ref	Ref	189 (1.2)	Ref	Ref
Yes	140 (3.4)	1.45 (1.20–1.76)	1.15 (0.94–1.41)	87 (2.1)	1.68 (1.30–2.16)	1.28 (0.98–1.66)
**NDI**	P<0.0001^e^			p<0.0001^e^		
Quartile 1 (≤25%)	105 (1.7)	Ref	Ref	50 (0.8)	Ref	Ref
Quartile 2 (>25–50%)	112 (2.8)	1.23 (0.95–1.60)	1.15 (0.88–1.49)	57 (1.1)	1.31 (0.90–1.92)	1.15 (0.79–1.68)
Quartile 3 (>50–75%)	125 (2.8)	1.64 (1.27–2.12)	1.37 (1.06–1.79)	74 (1.7)	2.03 (1.42–2.91)	1.50 (1.04–2.16)
Quartile 4 (>75%)	147 (4.1)	2.40 (1.87–3.07)	1.74 (1.33–2.27)	93 (2.6)	3.18 (2.26–4.47)	1.93 (1.34–2.79)
Missing	5 (4.8)	2.76 (1.15–6.62)	2.07 (0.85–5.04)	2 (1.9)	2.32 (0.57–9.40)	1.84 (0.45–7.51)
**Underlying comorbidities**						
No underlying comorbidities	225 (2.3)	Ref	Ref	114 (1.2)	Ref	Ref
Any underlying comorbidities	269 (2.8)	1.11 (1.03–1.21)	1.07 (0.99–1.15)	162 (1.6)	1.13 (1.02–1.25)	1.07 (0.97–1.18)
**Specific conditions** ^**f**^						
Allergy	134 (2.8)	1.12 (0.97–1.30)	1.14 (0.98–1.32)	59 (1.3)	0.88 (0.70–1.10)	0.88 (0.70–1.11)
Asthma	76 (3.2)	1.27 (1.03–1.56)	1.22 (0.99–1.51)	46 (1.9)	1.37 (1.05–1.79)	1.26 (0.96–1.64)
Autoimmune	33 (2.2)	0.88 (0.63–1.22)	0.88 (0.63–1.23)	40 (1.5)	1.02 (0.77–1.37)	1.09 (0.81–1.45)
Diabetes	7 (2.7)	1.05 (0.50–2.23)	1.04 (0.49–2.19)	4 (1.5)	1.08 (0.41–2.88)	1.03 (0.38–2.74)
Hypertension	12 (3.5)	1.39 (0.79–2.45)	-^g^	7 (2.0)	1.44 (0.69–3.02)	-^g^
Pre-pregnancy BMI						
Normal/Underweight (BMI<25)	174 (2.1)	Ref	Ref	83 (1.0)	Ref	Ref
Overweight (BMI 25–29.9)	138 (2.8)	1.18 (1.04–1.34)	1.09 (0.96–1.23)	87 (1.7)	1.36 (1.18–1.58)	1.19 (1.03–1.37)
Obese (BMI≥30)	135 (3.1)	1.28 (1.12–1.46)	1.03 (0.97–1.10)	92 (2.1)	1.54 (1.34–1.78)	1.06 (0.99–1.14)

BMI, Body Mass Index; EHR, Electronic Health Record; NDI, Neighborhood Deprivation Index; PR, Prevalence Ratios.

^a^ Prevalence of COVID-19 within variable stratum.

^b^ Prevalence ratios adjusted by race/ethnicity, maternal age, and NDI. NDI quartiles were calculated from the NDI distribution among total invited to complete the survey.

^c^ For 206 (41.9%) COVID-19 was documented in the EHR.

^d^ For 206 (74.4%) COVID-19 was self-reported.

^e^ p-value from chi-square test of infection prevalence across variable strata.

^f^ Separate models run for each health condition to estimate the prevalence ratio between women with and without the specific health condition.

^g^ Model did not converge due to small cell counts.

The crude prevalence of self-reported COVID-19 was highest in women with asthma (3.2%), hypertension (3.5%), and obesity (3.1%) and lowest in women with none of these conditions (2.3%) (**Table 2**). After adjustment for age, race/ethnicity, and NDI, the prevalence of self-reported COVID-19 did not significantly differ between women with and without these underlying comorbidities (**Table 2**). Similar patterns were observed for EHR-documented COVID-19, with the exception of a marginally significant higher prevalence of COVID-19 in overweight women compared with normal/underweight women (adj-PR [95% CI]: 1.19 [1.03–1.37]). Obesity, however, was not associated with COVID-19 after adjustment for covariates.

Among survey respondents who were pregnant at the time of survey completion (n = 14,353), 4,630 (32.3%) reported having received a diagnostic test for SARS-CoV-2 during their pregnancy, and among those, 402 (8.7%) reported a positive test result (**Table 3**). The percentage of women who reported receiving a test was highest in the first trimester of pregnancy (44.9%) and among women of Hispanic (34.9%) and other/missing race/ethnicity (39.1%), women with commercial insurance (33.4%), and women with underlying comorbidities (32.8–39.3%). The frequency of COVID-19 testing was higher among women who completed the survey in 2021 than in 2020. Among women who reported being tested, test positivity was highest for women responding to the survey in late 2020 and early 2021 (9.3–13.2%), the youngest women (<25 years: 20.8%), Black (16.4%) and Hispanic (16.4%) women, women on government insurance (17.8%), women living in neighborhoods in the highest quartile of deprivation (16.8%), and women with obesity (12.4%) or hypertension (12.9%). Among women who reported living with someone who had or likely had COVID-19, 53.8% were tested for COVID-19 with a test positivity frequency of 53.7%.

**Table 3:** Frequency of self-reported receipt of COVID-19 test and test-positivity among women who were currently pregnant at time of survey completion.

	Received COVID-19 Test n (%)^a^	Positive COVID-19 Test, among Tested n (%)^b^
**Self-reported COVID-19**	345 (84.2)	274 (79.4)
**EHR-documented COVID-19**	230 (91.3)	221 (96.1)
**Pregnant**	4630 (32.3)	402 (8.7)
**Trimester at time of survey completion**	p<0.0001	p = 0.051
** **1^st^ trimester	507 (44.9)	46 (9.1)
** **2^nd^ trimester	3546 (35.9)	291 (8.2)
** **3^rd^ trimester	577 (17.3)	65 (11.3)
**Month of Survey Completion**	p<0.0001	p<0.0001
June 2020	233 (9.1)	24 (10.3)
July	507 (14.1)	36 (7.1)
August	177 (22.0)	13 (7.3)
September	275 (30.4)	15 (5.5)
October	280 (38.3)	17 (6.1)
November	309 (43.2)	12 (3.9)
December 2020	453 (54.3)	42 (9.3)
January 2021	668 (60.3)	88 (13.2)
February	552 (60.3)	53 (9.6)
March	587 (62.7)	62 (10.6)
April	511 (60.0)	34 (6.7)
May 2021	78 (57.8)	6 (7.7)
**Maternal age (years)**	p = 0.004	p<0.0001
<25	197 (31.3)	41 (20.8)
25–29	761 (30.7)	123 (16.2)
30–34	1881 (32.6)	130 (6.9)
35+	1790 (34.4)	108 (6.0)
Missing	1 (16.7)	0 (0.0)
**Maternal race/ethnicity**	p = 0.0005	p<0.0001
Asian	1028 (30.9)	51 (5.0)
Black	165 (29.1)	27 (16.4)
Hispanic	1043 (34.9)	171 (16.4)
Pacific Islander/American Indian	64 (32.5)	9 (14.1)
Other/Missing	185 (39.1)	15 (8.1)
White	2145 (32.8)	129 (6.0)
**Insurance status**	p<0.0001	p<0.0001
Commercial	4363 (33.4)	358 (8.2)
Government	208 (24.9)	37 (17.8)
Unknown	59 (29.1)	7 (11.9)
**Neighborhood Deprivation Index** ^**d**^	p = 0.004	p<0.0001
Quartile 1 (≤25%)	1434 (33.0)	68 (4.7)
Quartile 2 (>25–50%)	1264 (33.0)	77 (6.1)
Quartile 3 (>50–75%)	1035 (32.3)	110 (10.6)
Quartile 4 (>75%)	848 (32.4)	142 (16.8)
Missing	49 (52.1)	5 (10.2)
**Underlying comorbidities**	p = 0.005^c^	p = 0.151^c^
No underlying comorbidities	2266 (31.2)	183 (8.1)
Any underlying comorbidities	2364 (33.4)	219 (9.3)
** **Allergy	1070 (34.8)	87 (8.1)
** **Asthma	591 (35.8)	54 (9.1)
** **Autoimmune	662 (34.1)	50 (7.6)
** **Obesity (BMI≥30)	1034 (32.8)	128 (12.4)
** **Diabetes	66 (39.3)	5 (7.6)
** **Hypertension	93 (34.8)	12 (12.9)
**Someone in household had or probably had COVID-19**	568 (53.8)	305 (53.7)

^a^ Proportion of respondents in stratum (row) who self-reported receiving a COVID-19 diagnostic test. 138 women were missing data on self-reported testing and were excluded from the denominator.

^b^ Proportion who reported testing positive for SARS-CoV-2 infection among those who reported receiving a diagnostic test.

^c^ Chi-square comparing frequency between women with and without any underlying comorbidities.

^d^ NDI quartiles were calculated from the NDI distribution among total invited to complete the survey.

### Symptoms, consequent healthcare utilization, and potential sources of COVID-19 exposure

Women with diagnosed COVID-19 were more likely to report living with someone who had or probably had COVID-19 (49.6% for self-report, 64.1% for EHR-documented), than women in the no-COVID-19 group (3.2%) (**Table 4**). Women with diagnosed COVID-19 according to self-report or EHR documentation were more likely than women in the no-COVID-19 group to experience several different symptoms during pregnancy (**Table 4**), including fever (33.6% and 33.0%, respectively vs. 3.8%), cough (48.0% and 39.1%, respectively vs. 8.0%), headache (56.1% and 55.8%, respectively vs. 22.0%), and fatigue or excessive sleepiness (50.0% and 54.0%, respectively vs. 23.5%). Loss of smell or taste was common among women with COVID-19, occurring in 42.3% of self-reported and 54.0% of EHR-documented COVID-19, compared with 1.2% in the no-COVID-19 group. Approximately 20% of women in either COVID-19 group experienced no symptoms compared with 61% in the no-COVID-19 group.

**Table 4:** Self-reported symptoms during pregnancy, consequent healthcare use, and possible sources of COVID-19 exposures in the two weeks before symptoms started.

	No COVID-19^a^ (Reference group) (n = 14,915)	Self-reported COVID-19 (n = 494)	p-value^b^	EHR-documented COVID-19 (n = 276)	p-value^c^
	n (%)	n (%)		n (%)	
**Someone in household had or probably had COVID-19**	477 (3.2)	245 (49.6)	<0.0001	177 (64.1)	<0.0001
**Symptoms**					
Fever or chills	566 (3.8)	166 (33.6)	<0.0001	91 (33.0)	<0.0001
Cough	1186 (8.0)	236 (48.0)	<0.0001	108 (39.1)	<0.0001
Shortness of breath	1154 (7.7)	148 (30.0)	<0.0001	78 (28.3)	<0.0001
Sore throat	1137 (7.6)	173 (35.0)	<0.0001	79 (28.6)	<0.0001
Headache	3284 (22.0)	277 (56.1)	<0.0001	154 (55.8)	<0.0001
Muscle or body ache	1368 (9.2)	198 (40.1)	<0.0001	115 (41.7)	<0.0001
Runny nose	1676 (11.2)	201 (40.7)	<0.0001	107 (38.8)	<0.0001
Fatigue or excessive sleepiness	3506 (23.5)	247 (50.0)	<0.0001	149 (54.0)	<0.0001
Diarrhea, nausea, or vomiting	2254 (15.1)	147 (29.8)	<0.0001	92 (33.3)	<0.0001
Loss of sense of smell or taste	175 (1.2)	209 (42.3)	<0.0001	149 (54.0)	<0.0001
Itchy or red eyes	504 (3.4)	28 (5.7)	0.006	15 (5.4)	0.064
None of the above	9124 (61.2)	83 (16.8)	<0.0001	51 (18.5)	<0.0001
**Among those with any symptoms**	**N = 5669**	**N = 409**		**N = 224**	
** Occurred as a result of symptoms** ^**d**^					
** **Overnight hospitalization	0 (0.00)	9 (2.2)	<0.0001	4 (1.8)	<0.0001
** **In-person healthcare visit	368 (6.5)	66 (16.1)	<0.0001	32 (14.3)	<0.0001
** **Telehealth visit	1143 (20.2)	289 (70.7)	<0.0001	158 (70.5)	<0.0001
** **Self-isolated/quarantined at home	892 (15.7)	308 (75.3)	<0.0001	172 (76.8)	<0.0001
** **None of the above	3642 (64.2)	18 (4.4)	<0.0001	17 (7.6)	<0.0001
** Potential exposures in the 2 weeks before symptoms** ^**d**^					
** **Have contact with someone who tested positive for COVID19	73 (1.3)	191 (46.7)	<0.0001	123 (54.9)	<0.0001
** **Have contact with someone who likely had COVID-19	107 (1.9)	62 (15.2)	<0.0001	34 (15.2)	<0.0001
** **Travel to different states or countries	155 (2.7)	19 (4.7)	0.025	9 (4.0)	0.207
None of the above	5322 (93.9)	160 (39.1)	<0.0001	71 (31.7)	<0.0001

^a^ Self-reported “No” COVID-19 diagnosis and did not have a documented COVID-19 diagnosis nor a suspected COVID-19 diagnosis in their EHR.

^b^ Chi-Square or Fisher exact test comparing self-reported COVID-19 to Reference group.

^c^ Chi-Square or Fisher exact test comparing EHR-documented COVID-19 to Reference group.

^d^ The denominator is restricted to women who reported any symptoms. Women without symptoms were not asked this question.

Among women who experienced symptoms during pregnancy, those in the self-reported and EHR-documented COVID-19 groups were significantly more likely than those in the no-COVID-19 group to report having had an overnight hospitalization (2.2% and 1.8%, respectively vs. 0%) or an in-person (16.1% and 14.3%, respectively vs. 6.5%) or telehealth visit with their provider (70.7% and 70.5%, respectively vs. 20.2%), self-isolating as a result of their symptoms (75.3% and 76.8%, respectively vs. 15.7%), and having had contact with someone who tested positive (46.7% and 54.9%, respectively vs. 1.3%) or likely had COVID-19 (both 15.2% vs. 1.9%) (**Table 4**). However, over a third of women with COVID-19 reported not having a known exposure to someone who had or likely had COVID-19.

## Discussion

In a large, diverse population of pregnant women during the first year of the COVID-19 pandemic in Northern California, we found that women who were <25 years of age, Hispanic, living in neighborhoods with high deprivation, and who had experienced personal or a partner’s job loss or reduced employment hours had the highest prevalence of diagnosed COVID-19. Over 50% of women with EHR-documented COVID-19 reported loss of smell or taste, although a fifth of women reported feeling no symptoms during their infection. Fewer than 3% of women with diagnosed COVID-19 and symptoms were hospitalized, suggesting that most symptomatic infections were not severe. Over a third of women with diagnosed COVID-19 were not aware of having had exposure to someone who had or likely had COVID-19 in the two weeks before their symptoms started.

The prevalence of COVID-19 in our sample of survey responders is lower than the prevalence in pregnant women reported by studies conducted in clinical settings across the US. These studies differed from ours in that they sampled over shorter time periods and from universal testing programs of hospitalized women admitted for delivery [10–15] or for COVID-19 related symptoms [1, 16]. A prevalence of 15% was reported among women who were admitted for delivery during the early surge of the pandemic in New York City (March 22-April 4, 2020) [15], when New York’s documented prevalence of COVID-19 in the adult population was much higher than in California [29]. During periods of rapid community transmission in the spring of 2020, Texas, Illinois, Massachusetts, and Connecticut observed infection rates of 2.6–8% in pregnant women who were universally screened for COVID-19 at delivery [9, 12–14]. In April and May, before Southern California witnessed summer surges in COVID-19 transmission, only 1% of pregnant women universally screened at delivery at Kaiser Permanente Southern California tested positive for SARS-CoV-2 [11]. The relatively low prevalence reported in both the Southern and Northern California samples may reflect the early success of local public health mitigation efforts at the beginning of the pandemic. However, in our sample, the low prevalence may also reflect the limited testing availability in the early course of the pandemic when KPNC was following CDC recommendations to test only those with severe symptoms or known exposures. Partway through the study’s observation period, KPNC began universal testing for all patients who presented for scheduled cesarean in October 2020 and for all patients in labor and delivery in December 2020. Northern California also experienced a surge in COVID-19 transmission across November 2020-February 2021, reflected in an observable increase in COVID-19 screening and test-positivity among respondents participating in the survey after October 2020.

The prevalence of COVID-19, whether measured by self-report of a diagnosis made by a healthcare provider or a documented diagnosis in the EHR, was generally highest among women <25 years old; Hispanic women; and women who lived in neighborhoods with high deprivation. Analyses of self-reported SARS-CoV-2 testing also indicated sociodemographic disparities in test-positivity rate, despite SARS-CoV-2 testing frequency being relatively similar across sociodemographic groups. A study in the broader KPNC population also found that sociodemographic factors, particularly race/ethnicity, were stronger predictors of SARS-CoV-2 infection but less predictive of SARS-CoV-2 testing use [30]. In other states, younger age [9, 14] and other sociodemographic indicators, including receipt of government health insurance [12, 14], were associated with heightened risk of COVID-19 in pregnant women. Earlier studies conducted in different US healthcare systems also consistently demonstrate that pregnant Black and Hispanic women have higher rates of COVID-19 [2, 9, 12, 14, 17, 20, 31] and are more likely to have severe symptoms leading to hospitalization [1, 17]. These COVID-19-related health disparities further highlight the pandemic’s interaction with pre-existing social determinants of health, employment in essential high-risk sectors, and systemic racial health inequities in the US [32–34].

In our sample, women with underlying asthma, hypertension, and obesity had a slightly higher prevalence of COVID-19 than their counterparts without these conditions, though these differences were not statistically significant after adjustment for covariates. Previous studies based on universal COVID-19 testing of pregnant women at delivery have found that several conditions, including diabetes, chronic lung diseases, cardiovascular disease, and obesity, were associated with increased risk of severe COVID-19 illness [1, 3, 9, 10, 14, 35]. While the relationship between allergy and COVID-19 has not been scrutinized in pregnant women prior to this study, studies in general populations present mixed evidence, suggesting that more research is needed to understand how specific conditions, treatments, and their interactions may shape COVID-19 susceptibility [23, 27]. Though very few women were hospitalized for COVID-19 in our sample, other studies suggest that underlying comorbidities may put pregnant women at increased risk of COVID-19 complications requiring hospitalization [2, 16, 31].

Pregnancy induces several physiological changes, including phases of immunosuppression and altered cardiopulmonary function, that can increase pregnant women’s susceptibility to infectious disease [36] as well as increase frequency of fatigue and headaches. Further, the pro-inflammatory states that naturally occur during the first and third trimesters can exacerbate a woman’s COVID-19 illness, inducing a cytokine storm of acute inflammation that can adversely affect the short and long-term health of the woman and fetus [37]. While COVID-19-like symptoms were prevalent in women without diagnosed COVID-19, most symptoms, including COVID-19 hallmarks such as cough, headache, and loss of smell or taste [22], were markedly more common in women with COVID-19. Previous studies have found that, among hospitalized women with COVID-19, some symptoms, including cough, fever, and myalgia were less common in pregnant than non-pregnant women, in part because universal testing of pregnant women has led to increased detection of mild and asymptomatic cases [2, 3, 38]. Studies in universal testing settings have demonstrated that 65–94% of pregnant women with SARS-CoV-2 infection experience no symptoms [9–13, 15, 18]. In contrast, only 20% of women with recognized SARS-CoV-2 infection were asymptomatic in our sample, likely reflecting the local testing policies in the early months of the pandemic that prioritized those with symptoms and known exposures.

Our study had some notable limitations. Response rates to the survey were relatively low, raising concerns about non-response bias. In addition, sociodemographic factors associated with higher risk of COVID-19 were also found to be associated with survey non-response. Our survey sample was more likely to be aged 30 years or older and more likely to be White relative to the general KPNC population, which is broadly representative of the insured adult population in California [21]. This potential selection bias suggests that the sociodemographic disparities we observed may underestimate disparities in the general population of pregnant women in California and nationally. Though the rates of some co-morbidities such as asthma, pre-existing diabetes, and obesity were similar in our sample to estimates in nationally-representative samples of the pregnant population, our sample was on average older with a higher proportion of women with commercial health insurance than in the national population [39–42].

The strengths of the present study include the examination of both self-reported and EHR-documented COVID-19 from a general population of pregnant women, providing additional insight regarding infection risk, symptoms, consequent healthcare use, and possible exposures beyond what has been described in earlier studies of pregnant women, which predominantly included women admitted to the hospital for delivery or for COVID-19 complications [2, 10–15]. Women who self-reported COVID-19 but who had no documentation of infection in the EHR may have received testing outside of KPNC and not reported their findings to their healthcare provider. Further strengths include the study’s large sample size, diversity across sociodemographic characteristics and stages in pregnancy, and large window of observation over the first year of the pandemic.

In conclusion, while COVID-19 was recognized in a relatively small number of women in our sample overall, our study identified several subgroups that may be particularly vulnerable to infection and observed unique infection-related symptoms during pregnancy, low frequency of hospitalization but high frequency of self-isolation and use of telehealth visits, and a sizable proportion of women with no known exposures to infected contacts before the onset of their symptoms. It will be important to follow the health of these women and their children in future studies.

## Supporting information

S1 Appendix KPNC COVID-19 pregnancy survey. (DOCX)

S2 Appendix ICD-10 codes used to determine EHR-confirmed and EHR-suspected COVID-19 diagnoses. (DOCX)

S1 Table Characteristics of eligible women overall and by survey response status, Kaiser Permanente Northern California. (DOCX)
