# Supplementary material for: COVID-19 prevalence, symptoms, and sociodemographic disparities in infection among insured pregnant women in Northern California
Source: PLoS One. 2021 Sep 3;16(9):e0256891. doi: 10.1371/journal.pone.0256891 (PMC8415576; doi:10.1371/journal.pone.0256891)
Supplement: S1 Appendix — (DOCX) [file pone.0256891.s001.docx]

**S1 Appendix.** KPNC COVID-19 Pregnancy Survey

**KPNC COVID-19 Pregnancy Survey**

**INSTRUCTIONS:**

**This form has 4 sections:**

- **Section A: COVID-19 Infection**
- **Section B: Impacts of the COVID-19 Outbreak on You**
- **Section C: Impacts of COVID-19 Outbreak on Pregnancy – Current**
- **Section D: Impacts of COVID-19 Outbreak on Pregnancy – Recall**

**Please complete Sections A and B. If you are pregnant, please also complete Section C. If you had a child after January 1^st^, 2020, please also complete Section D.**

**These questions are about your experience during your pregnancy with COVID-19, or the coronavirus. For each question, do the best you can to remember the details requested.**

**SECTION A: COVID-19 Infection**

For the following questions, healthcare provider means a doctor, nurse practitioner, physician assistant or anyone you go to for medical care.

1. During your pregnancy, did a healthcare provider ever tell you that you have, or likely have, COVID-19 (Coronavirus)?

1 ❑ Yes

2 ❑ No

2. During your pregnancy, which of the following symptoms did you have since February 1, 2020? (Mark all that apply)

1 ❑ Fever or chills

2 ❑ Cough

3 ❑ Shortness of breath

4 ❑ Sore throat

5 ❑ Headache

6 ❑ Muscle or body aches

7 ❑ Runny nose

8 ❑ Fatigue or excessive sleepiness

9 ❑ Diarrhea, nausea, or vomiting

10 ❑ Loss of sense of smell or taste

11 ❑ Itchy/red eyes

12 ❑ None of the above…*Go to Question 3*

2A. Which of the following occurred as a result of your symptoms? *(Mark all that apply)*

1 ❑ I was kept overnight in a hospital because a healthcare provider thought I had COVID-19

2 ❑ I saw a healthcare provider in person, such as in a clinic, doctor’s office, urgent care, or Emergency Room (ER)/Emergency Department (ED)

3 ❑ I spoke to a healthcare provider over the phone, by email, or online

4 ❑ I self-isolated or quarantined at home

5 ❑ None of the above

2B. In the two weeks before you had symptoms, did you: *(Mark all that apply)*

1 ❑ Have contact with someone who tested positive for COVID-19

2 ❑ Have contact with someone who likely had COVID-19 (e.g., was not tested but had symptoms; was told by a healthcare provider that he/she likely had it)

3 ❑ Travel to a different state or country (please specify: ___________________)

4 ❑ None of the above

3. Have you had the nose swab test for the virus that causes COVID-19? *(Mark all that apply)*

1 ❑ No, I never tried to get tested

2 ❑ No, I tried to get tested but was not able to

3 ❑ Yes, and I am waiting for the results

4 ❑ Yes, and the test showed that I did not have it (“negative” test)

5 ❑ Yes, and the test showed that I did have it (“positive” test)

4. Have you had a blood test to see whether you already had the COVID-19 virus (“serology”)? *(Mark all that apply)*

1 ❑ No, I never tried to get tested

2 ❑ No, I tried to get tested but was not able to

3 ❑ Yes, and I am waiting for the results

4 ❑ Yes, and the test showed that I did not have it (“negative” test)

5 ❑ Yes, and the test showed that I did have it (“positive” test)

5. Has anyone else living in your home had, or probably had, COVID-19?

1 ❑ Yes

2 ❑ No

**SECTION B: Impacts of the COVID-19 Outbreak on You**

1. In what ways did the COVID-19 outbreak affect your overall healthcare during your pregnancy? *(Mark all that apply)*

1 ❑ I did not go to healthcare appointments because I was concerned about entering my healthcare provider’s office

2 ❑ My healthcare provider canceled appointments

3 ❑ My healthcare provider changed to phone or online visits

4 ❑ My healthcare provider told me to self-isolate or quarantine

5 ❑ None of these apply

2. Which of the following behaviors did you do less of during your pregnancy because of the COVID-19 outbreak? *(Mark all that apply)*

1 ❑ In-person contact with people inside the home (that is, you are quarantined separately from one or more family or household members)

2 ❑ In-person contact with family who live outside the home

3 ❑ In-person contact with friends

4 ❑ In-person contact with colleagues at work

5 ❑ In-person events in the community, including religious events

6 ❑ None of these apply

3. Which of the following behaviors did you change during your pregnancy because of the COVID-19 outbreak? *(Mark all that apply)*

1 ❑ Eat more home-cooked meals

2 ❑ Eat more takeout / delivered food

3 ❑ Get more physical exercise

4 ❑ Get less physical exercise

5 ❑ Spend more time outdoors in nature

6 ❑ Spend less time outdoors in nature

7 ❑ None of these apply

4. In what ways did the COVID-19 outbreak affect your employment during your pregnancy? *(Mark all that apply)*

1 ❑ I kept my job and moved to working remotely or from home

2 ❑ I am on extended paid leave from my job (paid time off, sick leave, family leave)

3 ❑ I lost my job permanently

4 ❑ I lost my job temporarily, or was not told for how long

5 ❑ I got a new job

6 ❑ I reduced my work hours

7 ❑ I increased my work hours

8 ❑ My job put me at increased risk of getting COVID-19

9 ❑ I laid off employees

10 ❑ I did not have a paying job before the COVID-19 outbreak

11 ❑ None of these apply

5. In what ways did the COVID-19 outbreak affect your spouse/partner’s work during your pregnancy? *(Mark all that apply)*

0 ❑ Not applicable – I do not have a spouse/partner…*Go to Question 6*

1 ❑ My spouse/partner moved to working remotely or from home

2 ❑ My spouse/partner lost his/her job permanently

3 ❑ My spouse/partner lost his/her job temporarily, or was not told for how long

4 ❑ My spouse/partner got a new job

5 ❑ My spouse/partner reduced work hours

6 ❑ My spouse/partner increased work hours

7 ❑ My spouse/partner’s job puts him/her at increased risk of getting COVID-19

8 ❑ My spouse/partner laid off employees

9 ❑ My spouse/partner did not have a paying job before the COVID19 outbreak

10 ❑ None of these apply

6. How did the COVID-19 outbreak affect your regular childcare during your pregnancy? *(Mark all that apply)*

1 ❑ I had difficulty arranging for childcare

2 ❑ I had to pay more for childcare

3 ❑ My spouse/partner or I had to change our work schedule to care for our children ourselves

4 ❑ My regular childcare has not been affected by the COVID-19 outbreak

5 ❑ I do not have a child in childcare

7. What were your greatest sources of stress from the COVID-19 outbreak during your pregnancy? *(Mark all that apply)*

1 ❑ Health concerns

2 ❑ Financial concerns

3 ❑ Impact on work

4 ❑ Impact on your child

5 ❑ Impact on your community

6 ❑ Impact on family members

7 ❑ Access to food

8 ❑ Access to baby supplies (e.g. formula, diapers, wipes)

9 ❑ Access to personal care products or household supplies

10 ❑ Access to medical care, including mental health care

11 ❑ Social distancing or being quarantined

12 ❑ I am not stressed about the COVID-19 outbreak

8. What did you do to cope with your stress related to the COVID-19 outbreak during your pregnancy? *(Mark all that apply)*

1 ❑ Meditation and/or mindfulness practices

2 ❑ Prayer, religious services, and faith-based community support

3 ❑ Talking with friends and family (e.g., by phone, text, video, or social media posts)

4 ❑ Engaging in more family activities (e.g., games, sports)

5 ❑ Increased television watching or other “screen time” activities (e.g., video games, social media)

6 ❑ Eating more often, including snacking

7 ❑ Increasing time reading books, or doing activities like puzzles and crosswords

8 ❑ Drinking alcohol

9 ❑ Using tobacco (e.g., smoking, vaping)

10 ❑ Using marijuana (e.g., vaping, smoking, eating, dabbing)

11 ❑ Talking to my healthcare providers more frequently, include mental healthcare provider (e.g., therapist, psychologist, counselor)

12 ❑ Volunteer work

13 ❑ Exercised using online programs or videos (e.g., yoga, HIIT)

14 ❑ Went outside for a walk, run or bike ride

15 ❑ I have not done any of these things to cope with the COVID-19 outbreak

9. Please indicate the extent to which you view the COVID-19 outbreak as having either a positive or negative impact on your life during your pregnancy.

1 ❑ Extremely negative

2 ❑ Moderate negative

3 ❑ Somewhat negative

4 ❑ No impact

5 ❑ Slightly positive

6 ❑ Moderately positive

7 ❑ Extremely positive

10. Since becoming aware of the COVID-19 outbreak, how often did you feel happy and satisfied with your life during your pregnancy?

1 ❑ Not at all

2 ❑ Rarely

3 ❑ Sometimes

4 ❑ Often

5❑ Very often

11. Since becoming aware of the COVID-19 outbreak, you worried that your food would run out before you got money to buy more during your pregnancy.

1 ❑ Often true

2 ❑ Sometimes true

3 ❑ Never true

12. Since becoming aware of the COVID-19 outbreak, the food you bought just didn’t last and you didn’t have money to get more during your pregnancy.

1 ❑ Often true

2 ❑ Sometimes true

3 ❑ Never true

13. Since becoming aware of the COVID-19 outbreak, did **your household** receive benefits from any of the following food and nutrition assistance programs during your pregnancy?

1 ❑ CalFresh or Supplemental Nutrition Assistance Program (SNAP)

2 ❑ Special Supplemental Nutrition Program for Women, Infants, and Children (WIC)

3 ❑ California Food Access Program (CFAP)

4 ❑ Other food and nutrition assistance programs not listed

5 ❑ Our household did not receive benefits from food and nutrition assistance programs

14. Since becoming aware of the COVID-19 outbreak, how often during your pregnancy did you…

|  | **Not at all** | **Rarely** | **Sometimes** | **Often** | **Very Often** |
| --- | --- | --- | --- | --- | --- |
| a. have difficulty sleeping | 1 | 2 | 3 | 4 | 5 |
| b. startle easily | 1 | 2 | 3 | 4 | 5 |
| c. have angry outbursts | 1 | 2 | 3 | 4 | 5 |
| d. feel a sense of time slowing down | 1 | 2 | 3 | 4 | 5 |
| e. feel in a daze | 1 | 2 | 3 | 4 | 5 |
| f. try to avoid thoughts and feelings about  COVID-19 | 1 | 2 | 3 | 4 | 5 |
| g. try to avoid reading or watching information  about COVID-19 | 1 | 2 | 3 | 4 | 5 |
| h. have distressing dreams about COVID-19 | 1 | 2 | 3 | 4 | 5 |
| i. feel distressed when you see something that  reminds you of COVID-19 | 1 | 2 | 3 | 4 | 5 |

15. Over the last 2 weeks, how often have you been bothered by the following problems?

|  | **Not at all** | **Several days** | **More than half the days** | **Nearly everyday** |
| --- | --- | --- | --- | --- |
| a. Little interest or pleasure in doing things | 0 | 1 | 2 | 3 |
| b. Feeling down, depressed, or hopeless | 0 | 1 | 2 | 3 |
| c. Trouble falling or staying asleep, or  sleeping too much | 0 | 1 | 2 | 3 |
| d. Feeling tired or having little energy | 0 | 1 | 2 | 3 |
| e. Poor appetite or overeating | 0 | 1 | 2 | 3 |
| f. Feeling bad about yourself, or that you are  a failure, or have let yourself or your family down | 0 | 1 | 2 | 3 |
| g. Trouble concentrating on things, such as  reading the newspaper or watching television | 0 | 1 | 2 | 3 |
| h. Moving or speaking so slowly that other  people could have noticed. Or the opposite –  being so fidgety or restless that you have  been moving around a lot more than usual | 0 | 1 | 2 | 3 |

16. Over the last 2 weeks, how often have you been bothered by the following problems?

|  | **Not at all** | **Several days** | **Over half the days** | **Nearly everyday** |
| --- | --- | --- | --- | --- |
| a. Feeling nervous, anxious, or on edge | 0 | 1 | 2 | 3 |
| b. Not being able to stop or control worrying | 0 | 1 | 2 | 3 |
| c. Worrying too much about different things | 0 | 1 | 2 | 3 |
| d. Trouble relaxing | 0 | 1 | 2 | 3 |
| e. Being so restless that it's hard to sit still | 0 | 1 | 2 | 3 |
| f. Becoming easily annoyed or irritable | 0 | 1 | 2 | 3 |
| g. Feeling afraid as if something awful might  happen | 0 | 1 | 2 | 3 |

17. During the past month, which of the following have you done to socialize with friends, family, or others? *(Mark all that apply)*

1 ❑ Attended a group exercise class online

2 ❑ Attended a religious service online

3 ❑ Attended a virtual birthday celebration online

4 ❑ Had a conference call with friends or family, but did not use video

5 ❑ Had a video conference call with friends or family

6 ❑ None of these apply

18. During the past 7 days, please record the number of days that you did each of the following activities. Also, record the average minutes per day that you did each activity on the days that you did that activity.

|  | **Number of Days**  *(select only one choice)* | **Minutes per Day**  *(select only one choice)* |
| --- | --- | --- |
| Mild exercise, walking fast enough to cause your heart rate to increase somewhat | ❑ None  ❑ 1 – 2 days  ❑ 3 – 4 days  ❑ 5 – 6 days  ❑ Every day | ❑ 0 – 9 min.  ❑ 10 – 19 min.  ❑ 20 – 29 min.  ❑ 30 – 59 min.  ❑ 60 min. or more |
| Moderate exercise, sports or other physical activity that caused your heart rate to increase somewhat **(other than walking)** | ❑ None  ❑ 1 – 2 days  ❑ 3 – 4 days  ❑ 5 – 6 days  ❑ Every day | ❑ 0 – 9 min.  ❑ 10 – 19 min.  ❑ 20 – 29 min.  ❑ 30 – 59 min.  ❑ 60 min. or more |
| Vigorous exercise, sports or other physical activity that caused you to work up a sweat or caused your heart rate to greatly increase | ❑ None  ❑ 1 – 2 days  ❑ 3 – 4 days  ❑ 5 – 6 days  ❑ Every day | ❑ 0 – 9 min.  ❑ 10 – 19 min.  ❑ 20 – 29 min.  ❑ 30 – 59 min.  ❑ 60 min. or more |

19. To route you through the remaining questions, please mark whether:

❑ you are currently pregnant…*Go to Section C*

❑ you had a pregnancy that ended after January 1, 2020…*Go to Section D*

**SECTION C: Impacts of the COVID-19 Outbreak on Pregnancy - Current**

The following questions are about your current pregnancy.

1. Which of the following changes have you experienced as a result of the COVID-19 outbreak? *(Mark all that apply)*

1❑ I changed from planning a vaginal birth to a C-section or labor induction

2❑ My planned C-section or labor induction was changed

3❑ I changed from planning a home birth to planning a hospital birth

4❑ I changed from planning a hospital birth to planning a home birth

5❑ My healthcare provider canceled some or all of my prenatal visits

6❑My healthcare provider canceled some or all of my prenatal classes

7❑ I had more prenatal visits

8❑ My prenatal visits changed from in-person to phone or telemedicine/video

9❑ Nothing changed in my prenatal care or birth plan

1. In general, how distressed are you about **changes to your prenatal care** due to the COVID-19 outbreak?

1❑ Not at all

2❑ Mildly

3❑ Moderately

4❑ Extremely

***[SKIP TO END]***

**SECTION D: Impacts of the COVID-19 Outbreak on Pregnancy - Recall**

The following questions are about your recent pregnancy.

1. Which of the following changes did you experience as a result of the COVID-19 outbreak? *(Mark all that apply)*

1 ❑ I changed from planning a vaginal birth to a C-section or labor induction

2 ❑ My planned C-section or labor induction was changed

3 ❑ I delivered in the hospital instead of at home

4 ❑ I delivered at home instead of in the hospital

5 ❑ My healthcare provider cancelled some or all of my prenatal visits

6 ❑ My healthcare provider cancelled some or all of my prenatal visits

7 ❑ My healthcare provider canceled some or all of my prenatal classes

8 ❑ I had more prenatal visits

9 ❑ My prenatal visits changed from in-person to phone or telemedicine/video

10 ❑ My support people (e.g., spouse/partner, family) were not be permitted to attend delivery or visit after delivery

11 ❑ I was separated from my baby immediately after delivery

12 ❑ I changed from planning to breastfeed to feeding only formula

13 ❑ I changed from planning to feed only formula to breastfeeding

14 ❑ I changed from planning to breastfeed and feed formula to only breastfeeding

15 ❑ Nothing changed in my prenatal care, birth or newborn plans

1. In general, how stressed were you about **changes to your birth and newborn experiences** due to the COVID-19 outbreak?

1❑ Not at all

2❑ Mildly

3❑Moderately

4❑ Extremely

**Acknowledgement:** The survey was adapted from the NIH Environmental Influences on Children’s Health Outcomes (ECHO) COVID-19 Questionnaire [2020] developed for the ECHO-wide Cohort Data Collection Protocol.
