# Supplementary material for: COVID-19 prevalence, symptoms, and sociodemographic disparities in infection among insured pregnant women in Northern California
Source: PLoS One. 2021 Sep 3;16(9):e0256891. doi: 10.1371/journal.pone.0256891 (PMC8415576; doi:10.1371/journal.pone.0256891)
Supplement: S2 Appendix — (DOCX) [file pone.0256891.s002.docx]

**S2 Appendix**. ICD-10 codes used to determine EHR-confirmed and EHR-suspected COVID-19 diagnoses

| **Outcome status** | **DXID Number** | **DXID Description** | **List of ICD-10 Codes** |
| --- | --- | --- | --- |
| EHR-confirmed COVID-19 | 12459078 | Coronavirus COVID-19 Disease | U07.1 |
|  | 12459074 | Coronavirus COVID-19 Pneumonia | U07.1, J12.89 |
|  | 12459108 | COVID-19 | U07.1 |
|  | 12459075 | Coronavirus COVID-19 Acute Bronchitis | U07.1, J20.8 |
|  | 12459082 | Hx Of Coronavirus COVID-19 Disease | Z86.19 |
|  | 12459076 | Coronavirus COVID-19 Lower Respiratory Infection | U07.1, J22 |
|  | 12459077 | Asymptomatic Coronavirus COVID-19 Disease | U07.1 |
|  | 12459073 | Coronavirus COVID-19 Acute Respiratory Distress Syndrome | U07.1, J80 |
| EHR-suspected COVID-19 | 12459081 | SCREENING FOR CORONAVIRUS COVID-19 DISEASE (Undetermined/missing test result) | Z11.59 |
|  | 12459080 | Exposure to Coronavirus COVID-19 | Z20.828 |
|  | 12459107 | Coronavirus COVID-19 Disease Counseling | Z71.89 |
|  | 12459083 | Coronavirus COVID-19 Disease, Person Under Investigation | Z20.828 |
|  | 12459126 | SCREENING FOR ASYMPTOMATIC CORONAVIRUS COVID-19 DISEASE (Undetermined/missing test result) | Z11.59 |
