# Supplementary material for: COVID-19 prevalence, symptoms, and sociodemographic disparities in infection among insured pregnant women in Northern California
Source: PLoS One. 2021 Sep 3;16(9):e0256891. doi: 10.1371/journal.pone.0256891 (PMC8415576; doi:10.1371/journal.pone.0256891)
Supplement: S1 Table — (DOCX) [file pone.0256891.s003.docx]

**S1 Table.** Characteristics of eligible women overall and by survey response status, Kaiser Permanente Northern California

|  | **Total invited (n=82482)** | **Survey Respondents^a^**  **(n=19458)** | **Survey Non-respondents**  **(n=63024)** | **p-value^b^** |
| --- | --- | --- | --- | --- |
|  | **n (%)** | **n (%)** | **n (%)** |  |
| **Currently pregnant** | 59050 (71.6) | 14353 (73.8) | 44697 (70.9) | <0.0001 |
| **Postpartum** | 23432 (28.4) | 5105 (26.2) | 18327 (29.1) |  |
| **Maternal age** |  |  |  |  |
| <25 | 7116 (8.6) | 817 (4.2) | 6299 (10.0) | <0.0001 |
| 25-29 | 18297 (22.2) | 3257 (16.7) | 15040 (23.9) |  |
| 30-34 | 30388 (36.8) | 7788 (40.0) | 22600 (35.9) |  |
| 35+ | 26241 (31.8) | 7589 (39.0) | 18652 (29.6) |  |
| Missing | 440 (0.5) | 7 (0.04) | 433 (0.7) |  |
| **Maternal race/ethnicity** |  |  |  |  |
| Asian | 20663 (25.1) | 4543 (23.4) | 16120 (25.6) | <0.0001 |
| Black | 5970 (7.2) | 780 (4.0) | 5190 (8.2) |  |
| Hispanic | 22266 (27.0) | 4045 (20.8) | 18221 (28.9) |  |
| Pacific Islander/American Indian | 1495 (1.8) | 285 (1.5) | 1210 (1.9) |  |
| Other/Missing | 3146 (3.8) | 594 (3.1) | 2552 (4.1) |  |
| White | 28942 (35.1) | 9211 (47.3) | 19731 (31.3) |  |
| **NDI^c^** |  |  |  |  |
| Quartile 1 (≤25%) | 20312 (24.6) | 6085 (31.3) | 14227 (22.6) | <0.0001 |
| Quartile 2 (>25-50%) | 20393 (24.7) | 5278 (27.1) | 15115 (24.0) |  |
| Quartile 3 (>50-75%) | 20363 (24.7) | 4428 (22.8) | 15935 (25.3) |  |
| Quartile 4 (>75%) | 20379 (24.7) | 3562 (18.3) | 16817 (26.7) |  |
| Missing | 1035 (1.3) | 105 (0.5) | 930 (1.5) |  |
| **Underlying comorbidities** |  |  |  |  |
| Any underlying comorbidities | 42050 (51.0) | 10121 (52.0) | 31929 (50.7) | 0.010 |
| Allergy | 18966 (23.0) | 4720 (24.3) | 14246 (22.6) | <0.0001 |
| Asthma | 9720 (11.8) | 2381 (12.2) | 7339 (11.6) | 0.025 |
| Autoimmune | 10788 (13.1) | 2757 (14.2) | 8031 (12.7) | <0.0001 |
| Diabetes | 1188 (1.4) | 261 (1.3) | 927 (1.5) | 0.185 |
| Hypertension | 1299 (1.6) | 344 (1.8) | 955 (1.5) | 0.013 |
| Pre-pregnancy BMI |  |  |  |  |
| Normal/Underweight (BMI<25) | 31664 (38.4) | 8267 (42.5) | 23397 (37.1) | <0.0001 |
| Overweight (BMI 25-29.9) | 20847 (25.3) | 4999 (25.7) | 15848 (25.2) | <0.0001 |
| Obese (BMI≥30) | 19544 (23.7) | 4330 (22.3) | 15214 (24.1) | <0.0001 |
| **COVID-19 diagnosis documented in EHR** | 1944 (2.4) | 449 (2.3) | 1495 (2.4) | 0.604 |

EHR, Electronic Health Record; NDI, Neighborhood Deprivation Index.

^a^ Seventy-nine percent of respondents were KPNC members for at least 9 months in each of the two years preceding their survey completion date.

^b^ p-value from chi-square comparison between respondents and non-respondents.

^c^ NDI quartiles were calculated from the NDI distribution among total invited.
